# Adherence to and acceptability of home fortification with vitamins and minerals in children aged 6 to 23 months: a systematic review

**DOI:** 10.1186/s12889-016-2978-0

**Published:** 2016-04-07

**Authors:** Samara Fernandes de Barros, Marly Augusto Cardoso

**Affiliations:** Department of Nutrition, School of Public Health, University of São Paulo, Avenida Dr. Arnaldo 715, São Paulo, SP 01246-904 Brazil

**Keywords:** Multiple micronutrients in powder, Complementary feeding, Home fortification

## Abstract

**Background:**

Vitamin and mineral deficiencies affect more than two million people worldwide. In 2011, based on recent scientific evidence and the low effectiveness of current strategies, the World Health Organization recommended home fortification of foods with multiple micronutrients in powder (MNP) as a new strategy to prevent and control anaemia during childhood. This systematic review assessed adherence to and acceptability of home fortification with multiple micronutrients in powder (MNP) in complementary feeding.

**Methods:**

Adherence was assessed based on number or percentage of prescribed sachets that were consumed, and acceptability was assessed according to perceptions of caregivers and children about MNP.

**Results:**

In summary, the studies indicated that home fortification with MNP has good adherence, ranging from 50 % to over 90 % of the prescribed sachets and that MNP was well accepted by caregivers. Caregivers reported side effects in 3 % to 32 % of children taking MNP in many studies; diarrhoea, vomiting, and constipation were the most common.

**Conclusions:**

Home fortification with MNP has good adherence and acceptability in infants, with higher adherence in non-daily or flexible administration regimens. Characteristics of the target population and increased diarrhoea burden should be considered for planning public health programs with long term use of MNP. Acceptability of the MNP is satisfactory, when the use and perceived beneficial effects on children’s health are considered.

## Background

Vitamin and mineral deficiencies affect more than two million people worldwide [1]. Young children are highly vulnerable because their high nutrient requirements need to be achieved through diet, which is sometimes inadequate in quantity and quality of nutrients, especially in developing countries.

Iron deficiency anaemia is the most common preventable nutritional problem among young children, affecting more than 750 million children worldwide [2]. According to the World Health Organization, the prevalence of iron deficiency among young children is 2.5 times the prevalence of anaemia [3]. In 2011, based on recent scientific evidence and the low effectiveness of current strategies, the World Health Organization recommended home fortification of foods with multiple micronutrients in powder (MNP) as a new strategy to prevent and control anaemia during childhood [4]. Several formulations with different compositions of micronutrients are available for use in complementary feeding. For example, Sprinkles is the most used MNP and its formulation of micronutrients was first developed in 1996 by researchers at the Children’s Hospital in Toronto, to prevent micronutrient deficiencies in children [5].

Previous studies have described the efficacy of home fortification with MNP in different contexts, especially in low-income countries, where nutritional deficiencies in childhood are more prevalent. Most of these studies used increased average haemoglobin concentration as the main study outcome. However, few studies have evaluated adherence and acceptability, and when this information is available, adherence was assessed mainly by the number of sachets used. A recent systematic review of the effectiveness of MNP interventions found high acceptability with variable adherence, indicating that high acceptability does not always translate into adherence [1]. Adherence in trials has been defined as the extent to which a patient follows advice regarding the use of a supplement, for example. Studies have found that high adherence with fortified complementary foods has often been reflected in improved nutritional status, suggesting that adherence is a key factor in determining the effectiveness of the fortification strategy for MNP interventions [5, 6].

It is known that adherence and acceptability are key factors in the success of an intervention. Thus, this systematic review aimed to summarize the scientific literature examining the adherence to and acceptability of home fortification with MNP in complementary feeding for planning and evaluation of public health programs to prevent childhood anemia.

## Methods

We performed a systematic review of the scientific literature in December 2013, searching for indexed published articles in the electronic databases of the U.S. National Library of Medicine and the National Institutes of Health (PubMed), the Latin American and Caribbean Literature on Health Sciences (LILACS), the Scientific Electronic Library Online (SciELO), and Thomson Reuters IP & Science (Web of Knowledge).

The descriptors used to select articles were “micronutrients powder” OR “home fortification” OR “Sprinkles.” Truncation symbols were used with the searched terms in Spanish and English to find words with the same root, increasing the chances of detecting a greater number of items. The selection of papers was restricted to articles published from 2003 to 2014 and written in Portuguese, Spanish, or English. The reference lists and some potentially relevant articles cited were examined and read to find additional articles or important information to the review.

The main eligibility criteria for selection of the published studies included a focus on children aged 6 months to 2 years or more and available information on adherence to and/or acceptability of home fortification of complementary feeding with MNP. Studies with either qualitative and/or quantitative methods were accepted.

Studies that used multiple micronutrients not in powder form, articles with target populations not in the age range of interest, abstracts or Congress Annals, other review articles, and technical reports were excluded for the purpose of this review.

The identification and selection of the articles were performed by two authors independently. Studies were initially selected based on their titles, and these were included in the next step of reading abstracts. Articles selected based on their abstracts were then read in full before the final selection decision, based on the criteria listed above. Discrepancies were resolved by the two authors.

**Fig. 1:**
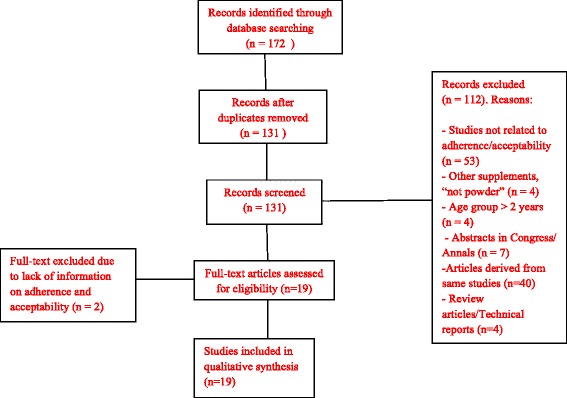
Flowchart of article selection

A flowchart for processing and data analysis was organized to describe the steps in paper selection (Fig. 1). After all papers were read, a spreadsheet was created in Microsoft Excel 2010 to extract the relevant information from each study. Virtual Endnote Web software was used to assist in organizing the articles and writing this manuscript. Finally, descriptive tables were created with a synthesis of included articles, listing the following information: author, publication year, country, prevalence of nutritional disorders, study aim, population and design, intervention characteristics and results regarding adherence, acceptability, and side effects of home fortification with multiple micronutrients. Considering the scope of this review with a focus on available data on adherence to and acceptability for the use of MNP in complementary feeding, we did not follow strict criteria for assessing quality of the revised articles. In this review, adherence was defined as a quantitative measure of the number of sachets used compared with the expected use over a specified time frame informed in the revised studies; acceptability was considered as a subjective measure related to caregivers perceptions of the child’s acceptance of the sachet, as well as perceptions of associated health benefits or side effects.

## Results

Overall, 131 references were found using our search criteria. Most articles were excluded after reading their titles and abstracts based on the exclusion criteria, leaving only 19 articles eligible for full reading. Of these, two were excluded because they did not provide information on adherence to and acceptability of home fortification. The remaining 17 articles that met the inclusion criteria were selected for detailed analysis of adherence to and acceptability of home fortification with MNP in complementary feeding (Fig. 1).

Tables 1 and 2 shows the main characteristics of the selected 14 clinical trials and three observational studies, respectively. Most of these studies were conducted in low-income countries: two in South America [7, 8], one in Central America [9], three in North America [10–12], eight in Asia [13–20], and three in Africa [21–23].

**Table 1:** Clinical trials assessing adherence to and acceptability of home fortification with multiple micronutrients in powder (MNP) in complementary feeding

Source study (*n* = 14)	Setting	Prevalence of nutritional disorders	Study aim	Study design	Sample at baseline	Intervention
Soofi et al. (2013) [20]	Sindh, Pakistan (urban and rural areas)	Stunting = 23.3 %, 30.1 %, and 26.9 %; anaemia = 17.1 %, 26.6 %, and 24.9 %, in control, MNP with zinc, and MNP without zinc groups, respectively.	To assess the effects of provision of two MNP formulations, with or without zinc, on children’s growth, micronutrient status, and morbidity.	Cluster-randomized trial. Three groups: non-supplemented control, MNP without zinc, and MNP with 10 mg of zinc.	*n* = 2746 children, aged 6 months.	Frequency of MNP: daily Intervention duration: 1 year Total sachets prescribed: 360 Iron dosage in MNP: no data given
Jack et al. (2012) [17]	Cambodia	Stunting = 11.3 % and 13.3 %; anaemia = 83.7 % and 84.4 %, in control and intervention groups, respectively.	To evaluate the effect of MNP alongside infant and young child feeding education program compared with feeding education alone on anaemia, iron deficiency, vitamin A, zinc, and growth in Cambodian infants.	Cluster-randomized effectiveness trial. Two groups: non-supplemented control received infant and young child feeding education alone; supplemented group received MNP alongside education.	*n* = 1350 children, aged 6 months.	Frequency: daily Intervention duration: 6 months Total sachets prescribed: 180 Iron dosage in MNP: 12.5 mg
Inayati et al. (2012) [15]	Nias Island, Indonesia	Stunting = 34.1 %; anaemia = 51.7 %.	To assess the impact of intensive nutrition education with or without the provision of MNP on the nutritional status of mildly wasted children.	Cluster-randomized trial. Four groups: MNP plus intensive education; intensive nutritional education alone; supplemented MNP plus monthly non-intensive nutrition education program; non-intensive education program alone.	*n* = 215 children, aged 6 to 60 months.	Frequency: daily Intervention duration: 1 year (or until child reached a weight-for-height z score ≥ -1) Total sachets prescribed: 360 Iron dosage in MNP: 10 mg
Sampaio et al. (2012) [7]	Bahia, Brazil	Stunting = 5.3 % and 7.4 %, respectively, in intervention and control groups.	To evaluate the incidence of diarrheal disease and acute respiratory infection in children undergoing supplementation with zinc and other micronutrients through the use of MNP.	Randomized clinical trial, double blind. Two groups: supplemented control with MNP without zinc; intervention group supplemented with MNP with zinc.	*n* = 143 children, aged 6 to 48 months.	Frequency: daily Intervention duration: 90 days Total sachets prescribed: 90 Iron dosage in MNP: 12.5 mg
Avula et al. (2011) [13]	Rajasthan, India	Stunting = 48.0 % in India.	To assess the impact of the existing Supplemental Nutrition Program with local production of supplemental food, home fortification with MNP, and monitoring.	Quasi-experimental. Two groups: control group received the usual Supplementation Nutrition Program; intervention group received the enhanced program.	*n* = 1128 children, aged 6 to 30 months.	Frequency: five times weekly Intervention duration: 6 months Total sachets prescribed: ≈ 120 Iron dosage in MNP: 12 mg
Kounnavong et al. (2011) [18]	Rural community in Lao People’s Democratic Republic	Stunting = 44.5 %, 42.3 %, and 40.4 % in control, twice weekly, and daily groups, respectively. Anaemia = 63.5 %.	To compare the effect of twice weekly versus daily supplementation with MNP on anaemia prevalence, haemoglobin concentration, and growth in infants and young children.	Randomized trial. Three groups: non-supplemented control, twice weekly supplementation, and daily supplementation.	*n* = 336 children, aged 6 to 52 months.	Frequency: daily or twice weekly. Intervention duration: 24 weeks Total sachets prescribed: 168 (daily group) or 48 (twice weekly) Iron dosage in MNP: 10 mg
Tripp et al. (2011) [23]	Niger	Stunting = 49.0 %; Anaemia = 91.0 %.	To assess the acceptability of an MNP and a lipid-based nutrient supplement (Nutributter), and to explore people’s willingness to pay for these products.	Qualitative study. Two groups: 1. Supplemented with MNP or Nutributter for 4 weeks; 2. Supplemented with MNP and Nutributter for 2 weeks each.	*n* = 83 children, aged 6 to 23 months.	Frequency: daily Intervention duration: 4 weeks Total sachets prescribed: 28 or 14 Iron dosage in MNP: 6 mg
Lundeen et al. (2010) [19]	Kyrgyz Republic	Anaemia = 50.0 %.	To test the effectiveness of a 2-month intervention with daily home fortification of complementary food using MNP in reducing anaemia among children 6 to 36 months of age.	Cluster-randomized trial. Two groups: non-supplemented control and intervention supplemented with multiple micronutrients.	*n* = 2193 children, 6 to 36 months.	Frequency: daily Intervention duration: 2 months Total sachets prescribed: 60 Iron dosage in MNP: 12.5 mg
Rosado et al. (2010) [12]	Querétaro, México	No data given.	To evaluate the efficacy and children’s acceptance of several recognized strategies to treat anaemia.	Randomized clinical trial. Five groups: 1. Iron supplement, 2. Iron + folic acid supplement, 3. MNP, 4. Micronutrient-fortified complementary food as porridge powder, and 5. Zinc + iron + ascorbic acid-fortified water.	*n* = 266 children, aged 6 to 43 months.	Frequency: daily. Intervention duration: 4 months Total sachets prescribed: 120 Iron dosage in MNP: 10 mg
Geltman et al. (2009) [11]	United States	Iron deficiency = 15.0–35.0 %.	To determine whether low-income infants’ adherence to MNP was better than to ferrous sulfate drops.	Randomized clinical trial. Two groups: Supplemented with MNP, supplemented with iron syrup.	*n* = 150 children, aged 6 months.	Frequency: daily Intervention duration: 3 months Total sachets prescribed: 90 Iron dosage in MNP: 12.5 mg Iron dosage in ferrous sulphate drops: 10 mg of elemental iron
Adu-Afarwuah et al. (2008) [21]	Ghana	Anaemia = 23.0–30.0 % in both groups.	To compare the efficacy and acceptability of MNP, Nutritabs, and fat-based Nutributter, which provide 6, 16, and 19 vitamins and minerals, respectively, when used for home fortification of complementary foods.	Randomized trial. Four groups: non-supplemented; supplemented with MNP; supplemented with Nutritabs; supplemented with Nutributter.	*n* = 313 children, aged 6 months.	Frequency: daily Intervention duration: 6 months Total sachets prescribed: 180 Iron dosage in MNP: 12.5 mg
Ip et al. (2007) [16]	Bangladesh	Anaemia = 75.8 %, 81.7 %, and 73.0 %, in daily for 2 months, flexible for 3 months, and flexible for 4 months groups, respectively.	To compare the effects of daily versus flexible administration of MNP on adherence, acceptability, and haematological status among young children in rural Bangladesh.	Cluster-randomized trial Three groups: supplemented daily with MNP for 2 months; supplemented with MNP with a flexible regimen for 3 months; supplemented with MNP with a flexible regimen for 4 months.	*n* = 362 children, aged 6 to 24 months.	Frequency: Depended on the treatment regimen. The children received 60 sachets each. Intervention duration: 2, 3, or 4 months. Total sachets prescribed: 60 Iron dosage in MNP: 12.5 mg
Menon et al. (2007) [9]	Haiti	Anaemia = 52.0 % and 37.0 % in groups 1 and 2, respectively.	To evaluate the effectiveness of 2-months treatment with MNP in reducing anaemia among children 9–24 months.	Cluster-randomized pre-post intervention trial. Two groups: group 1 supplemented with MNP plus fortified food; group 2 supplemented with fortified food alone.	*n* = 41 children, aged 9 to 24 months.	Frequency: daily Intervention duration: 2 months Total sachets prescribed: 60 Iron dosage in MNP: 12.5 mg
Christofides et al. (2005) [10]	Aboriginal communities in Canada	Anaemia = 36.0 %.	To determine the acceptability and safety of MNP as a strategy for delivering iron to infants and young children	Double-blinded randomized controlled trial. Two groups: control; supplemented with MNP.	*n* = 102 children, aged 4 to 18 months.	Frequency: daily Intervention duration: 6 months Total sachets prescribed: 180 Iron dosage in MNP: 30 mg

**Table 2:** Observational studies assessing adherence to and acceptability of home fortification with multiple micronutrients in powder (MNP) in complementary feeding

Source study (*n* = 3)	Site	Prevalence of nutritional disorders	Study aim	Study design	Sample at baseline	Intervention
Espino et al. (2012) [8]	Apurímac, Peru	Anaemia = 64.0 %.	To evaluate the implementation of the program of universal supplementation with MNP “chispitas” through the quantity and quality of sachets consumed and its relation to anaemia.	Cluster-randomized trial. One group: supplemented with MNP	*n* = 714 children, aged 6 to 35 months.	Frequency: at least 15 sachets monthly Intervention duration: 6 months Total sachets prescribed: 90 Iron dosage in MNP: 12.5 mg
Bilukha et al. (2011) [14]	Butan	Stunting = 39.2 %; anemia = 43.3 %.	To evaluate the effectiveness of a program to distribute MNP on a large scale in reducing the prevalence of anaemia and monitoring morbidity and growth in refugee children.	Longitudinal cluster-randomized trial. One group: supplemented with MNP (no control group).	*n* = 502 children, aged 6 to 59 months.	Frequency: 15 sachets monthly Intervention duration: 26 months Total sachets prescribed: 390 Iron dosage in MNP: 10 mg
Jefferds et al. (2010) [22]	Kenya	No data given.	To describe community members’ reactions to and experiences using MNP, with an emphasis on acceptability, utilization, and promotion.	Qualitative study.	*n* = 47 children, aged 6 to 59 months.	Frequency: daily Intervention duration: 1 month Total sachets prescribed: 30 Iron dosage in MNP: 12.5 mg

A high prevalence of anaemia and stunting was found in children less than 2 years of age at analysed sites. The sample size in these studies ranged from 47 to 2,746 children and the duration of intervention ranged from 1 to 26 months, with a 6-month intervention in six studies [8, 10, ,1317,18,21]. Twelve studies offered MNP to children daily [7, 9–12, 15, 17, 19–23], three adopted non-daily administration, five or fewer sachets per week [8, 13, 14] and two articles compared daily with non-daily administration [16, 18]. In addition, five studies compared MNP with other supplement types or fortified foods [9, 11, 12, 21, 23] and two studies compared different MNP formulations [7, 20].

In all reviewed studies caregivers received instructions about appropriate use of the MNP sachets. One package of MNP should be added in a small portion of usual semi-solid food to be consumed fully for the child. Only in study conducted by Sampaio et al. MNP sachets were administered out of home, in childcare centre. The authors commented that the banana was the food commonly used to mix MNP sachets; in remaining studies no information on this was provided [7].

Table 3 summarizes the information collected on adherence to and acceptability of home fortification with MNP in young children. Reviewed studies assessed adherence based on number or percentage of prescribed sachets that were consumed. Acceptability was assessed according to perceptions of caregivers and children about MNP. For example, the reasons for children or caregivers like or dislike of MNP, such as taste, flavor, ease of use, ease of transportability, health benefits and others.

**Table 3:** Summary of results regarding adherence to and acceptability and side effects of home fortification with multiple micronutrients in powder (MNP)

Source study (*n* = 17)	Adherence (or percentage of use) and acceptability	Side effects and/or limitations
Soofi et al. (2013) [20]	Adherence: The mean number of micronutrient powder sachets consumed each month was 16.8 (SD 11.7) in the MNP without zinc group and 15.2 (11.9) in the MNP with zinc group. Acceptability: No data given.	Side effects: Increased proportion of days with diarrhoea in MNP without zinc group (OR 1.15; 95 % CI 1.00–1.33) and in MNP with zinc group (OR 1.31; 95 % CI 1.13–1.51), *p* = 0.001. Increased incidence of bloody diarrhoea in MNP without zinc group (IRR 1.63; 95 % CI 1.12–2.39) and in MNP with zinc group (IRR 1.88; 95 % CI 1.29–2.74), *p* = 0.003.
Espino et al. (2012) [8]	Adherence: 5.4 % of children did not receive the intervention; 60.3 % consumed 60 or more sachets and only 49.0 % consumed them adequately (sachets consumed fully and with semisolid food). Acceptability: Among those who received the intervention, 4.5 % reported that they stopped giving sachets to children (of these, 70.0 % of the children not want to eat food with sachets) and 30.4 % of children did not consume the sachets adequately (of these, 84.0 % did not like the MNP flavor).	Side effects: No data given.
Jack et al. (2012) [17]	Adherence: 93.3 % of eligible children used MNP; the median number of MNP sachets consumed per month per child was 23.8 (range, 0–30). Acceptability: No data given	Side effects: No data given.
Inayati et al. (2012) [15]	Adherence: The proportion of children who consumed MNP daily was higher in the intensive nutrition education + MNP group than in the non-intensive nutrition education + MNP group (83.0 % *vs* 62.0 %). Acceptability: The main reasons given for not regularly consuming MNP included: perceived bitter taste of foods when mixed with MNP, monotonous taste when consumed daily, and occasionally forgetting to add MNP supplement to the lunch of a mildly wasted child. However, the majority of caregivers stated that they regularly added the MNP to the child’s meal, but the child several times refused to consume it (data not shown).	Side effects: No data given.
Sampaio et al. (2012) [7]	Adherence: The mean percentage of consumption, in days, of the entire contents of the sachets was 95.7 % (SD = 4.9) in the test group supplemented with MNP with zinc, and 96.4 % (SD = 6.2) in the control group supplemented with MNP without zinc. Acceptability: No data given	Side effects: No data given.
Avula et al. (2011) [13]	Adherence: No data given. Acceptability: No data given.	Side effects: In this qualitative study, two of the five health workers said that there were some complaints from mothers regarding instances of diarrhoea; when MNP were administered, the children started losing weight. No data shown for the control group.
Bilukha et al. (2011) [14]	Adherence: Over 90.0 % of children in each of the surveys from 2008 to 2010 were reported to currently consume MNP. Acceptability: 40.0 % of caregivers reported changes to food after mixing it with MNP. In 2010, 80.0 % to 85.0 % of caregivers reported perceived positive changes in children’s health after receiving MNP.	Side effects: The percentage of total of caregivers that reported any perceived negative health effects (diarrhoea, vomiting, constipation, etc.) attributed to multiple micronutrients was 11.6 % in 2008, 5.6 % in 2009, and 2.9 % in 2010. This study had no control group.
Kounnavong et al. (2011) [18]	Adherence: All children in the twice-weekly group consumed two sachets of MNP per week. In the daily group, 72.7 % of children consumed five or more sachets per week and 43.6 % consumed all seven sachets per week for all 24 weeks. Acceptability: 42.1 % of mothers reported that MNP changed the colour of their children’s food and 43.9 % reported that it had an unpleasant smell or taste. However, some mothers mixed the MNP in liquids such as juice or milk. Many of the mothers felt that the MNP had increased their child’s appetite (31.7 %) and playfulness (48.4 %).	Side effects: There were no significant differences in reports of illness (diarrhoea or cough) among the control, daily, and twice weekly groups (32.7 %, 39.1 %, and 34.2 %, respectively; *p* = 0.587).
Tripp et al. (2011) [23]	Adherence: No data given. Acceptability: MNP sachets were found to be acceptable and beneficial by mothers. Mothers said that MNP were easy to use, and several liked that the product had no taste or smell and did not change the taste of the food. Almost all mothers, regardless of the product they used, reported some increase in appetite or weight gain in their child (data not shown).	Side effects: Several mothers reported diarrhoea in their children at the start of the study (data not shown).
Jefferds et al. (2010) [22]	Adherence: At midway (at 2 weeks of 1 month-study), observations of sachets in 24 households showed an average of 15 sachets used per child per household (range, 5 to 25). Five families reported giving away MNP to children living in other households or older children taking and consuming MNP without permission. Acceptability: MNP’s acceptability was high and most families reported that the children ate food with MNP without problems. Perceived positive effects observed in children: increased appetite and improvements in immunity, strength, activity levels, and weight gain (data not shown).	Side effects: Infrequently mentioned by some caregivers were initial adjustments to MNP, including diarrhoea, softer stool, dark stool, and vomiting.
Lundeen et al. (2010) [19]	Adherence: Adherence was high. Average consumption of 45 of the 60 sachets provided; 39.0 % of the children consumed all 60 sachets. Acceptability: 67.0 % of caregivers reported that they liked using MNP, 73.0 % reported that the MNP was easy to use, and 57.0 % reported an improvement in their children’s appetite.	Side effects: Among participants in the intervention group, 32.0 % of caretakers reported diarrhoea on 3 or more days during the 2-month intervention, 29.0 % reported constipation, 9.0 % reported vomiting, 4.0 % reported an allergic reaction. No data shown for control group. Authors comment that these data must be cautiously interpreted because diarrhoea and other forms of gastrointestinal upset are quite common among young children in the Kyrgyz Republic.
Rosado et al. (2010) [12]	Adherence: 84.6 % (95 % CI, 71.9 %–97.2 %) of children in the MNP group completed 80.0 % of treatment dose (adequate adherence) and 71.0 % (95 % CI, 57.7 %–84.4 %) in this group completed the 4 months of treatment. Acceptability: In this study the MNP treatment had the lowest acceptability. In this group, 6.4 % (95 % CI, 5.5–7.2) of children had trouble taking the treatment and 7.4 % (95 % CI, 6.5–8.4) disliked the treatment.	Side effects: In the MNP group, the proportion of children experiencing any adverse event (allergies, infections, or viral diseases) was 10.9 %. In other groups this proportion was 4.3 % (iron supplement), 5.4 % (fortified food), 7.0 % (zinc and iron and acid ascorbic fortified water), and 4.9 % (iron and folic acid supplement).
Geltman et al. (2009) [11]	Adherence: High adherence (5 to 7 sachets consumed per week) ranged from 30.0 % to 46.0 % in the group supplemented with MNP. Acceptability: 12.0 % of caregivers in the MNP group reported concerns about using a new product, 14.0 % reported concerns about safety of the product for infants, and 17.0 % reported difficulty in integrating administration of the supplement into a daily routine.	Side effects: The main side effects in the MNP group were: constipation (15.0 % versus 26 % in iron drops group; *p* = 0.14); diarrhoea (11.0 % versus 12 % in iron drops group; *p* = 0.85), and vomiting (5.6 % versus 8.8 % in iron drops group; *p* = 0.51).
Adu-Afarwuah et al. (2008) [21]	Adherence: At 12 months, median adherence (95 % CI) to treatment in the MNP group was 85.8 % (82.3–90.0). This adherence was determined as the percentage of scheduled days on which the supplement was added to the child’s food. Acceptability: In the MNP group, 96.9 % of mothers liked giving the supplements to their children, 99.0 % of mothers believed that consumption of the supplements benefited their children’s health, 89.6 % of mothers said that the child easily accepted food mixed with the supplement, 95.9 % of mothers did not have major problems feeding the child the supplement, and 100.0 % of them had a good impression of the supplement.	Side effects: No significant side effects were reported (data not shown).
Ip et al. (2007) [16]	Adherence: On average, children in the flexible 4-months group consumed 98.0 % of prescribed sachets. The flexible 3-months group consumed an average of 93.0 % and the daily 2-months group consumed 88.0 %. The proportion of children who consumed all prescribed sachets was 86.4 %, 58.4 %, and 13.5 % in the flexible 4-months, flexible 3-months, and daily 2-months groups, respectively. Acceptability: Most mothers reported changes in their child’s behaviour (increased appetite and higher levels of activity and playfulness) after using MNP. MNPs were found to mix easily with food and had ‘no’ or ‘mild’ effects on the colour, taste, and smell of foods to which they were added. Almost all mothers preferred flexible administration over daily schedule (data not shown).	Side effects: No data given.
Menon et al. (2007) [9]	Adherence: An estimated mean 57.6 of the planned 60 sachets were consumed (SD, 4.9; range, 27–60). Acceptability: No data given.	Side effects: No data given.
Christofides et al. (2005) [10]	Adherence: Average adherence was 59.6 % (SD, 27.7). Adherence was determined by first calculating a score for each individual based on the mean outcome over 12 monitoring visits, and then calculating the overall mean percentage in the study population. Acceptability: Mothers said that the MNP did not create any appreciable change in colour, taste, or appearance of the complementary food and that they were easy to use, although they did have trouble remembering to give them daily. Participants agreed that if MNP were to become a commercially marketed product, they should be made available in their communities as an alternative to iron syrup.	Side effects: In the MNP group, the main side effects were diarrhoea (28.6 % versus 33.9 % in the placebo group; RR, 1.09; 95 % CI, 0.61–1.97) and vomiting (8.2 % versus 20.7 % in the placebo group; RR, 0.57; 95 % CI, 0.23–1.39).

*CI* confidence interval, *IRR* incidence rate ratio, *OR* odds ratio, *RR* relative risk, *SD* standard deviation

### Adherence and acceptability

Eight studies reported an average consumption of sachets ranging from 50 % to 96 % of the recommended number [7, 9, 10, 17, 19–22]. Only one study reported unsatisfactory adherence to MNP administration. Those authors considered consumption of five to seven sachets per week to be high adherence, and this number was achieved by 31–46 % of participants in the study [11].

In the two papers that assessed adherence to consumption of sachets in a daily versus a non-daily scheme, the authors found higher rates of adherence in non-daily regimes, and in these groups the average consumption was greater than 90 % of the prescribed sachets [16, 18].

In general, MNP were well accepted by caregivers and by children. Among the mentioned factors that contributed to high acceptability were transportability, ease of preparation and use, perceived benefits to children’s health (increased appetite and agility), and preservation of the organoleptic characteristics of the children’s meals. Nevertheless, some studies reported that MNP caused changes in the colour or taste of food, including yellowing of rice, bitterness of food after mixing with MNP, and the unpleasant taste of MNP itself. In most of these studies, the authors did not report whether the MNP had been consumed with liquids, such as broth soups or fruit juices [12, 14, 15, 18].

Most mothers expressed interest in continuing MNP use and recommended MNP to other mothers, if these products were available in their regions. In the study by Rosado et al. 2010 MNP had lower acceptability than other supplements used in the study (iron supplement, iron and folic acid supplement, micronutrient-fortified complementary food or zinc/iron/ascorbic acid fortified water). According to the authors, most cases of rejection resulted from the taste of the MNP, probably because of their mineral concentrations [12].

### Side effects

The most commonly reported side effects in these studies were diarrhoea, vomiting, and constipation, ranging from 3 % to 32 % of participants.

Changes in the colour of stools were seen in 95 % of participants in the study by Lundeen et al.; however, this is a harmless expected effect of iron supplementation. These authors did not comment on whether differences in adherence were associated with the occurrence of side effects [19].

## Discussion

This systematic review gathered seventeen papers and analysed the main conclusions from their results. Firstly, home fortification with MNP has good adherence in regards to the percentage of sachets consumed, with higher adherence in non-daily administration regimens. Secondly, acceptability of the MNP is satisfactory, when the use and perceived beneficial effects on children’s health are considered. However, limitations persist such as limited knowledge and experience of professionals and caregivers in the use of MNP, the resolution of which could increase MNP’s acceptability. Finally, MNP side effects ranged from 3 % to 32 % of participants, and in general posed a low risk to health.

Higher adherence in flexible administration regimens compared to daily administration can be attributed to mothers’ difficulty in remembering to offer sachets daily and the possibility that mothers discontinued administration when sachets were forgotten [10].

Adherence to non-daily regimens reached 100 % (all children consumed 100 % of prescribed MNP sachets) in the study conducted by Kounnavong et al. [18]; however, a comparison of the impact of non-daily administration versus daily administration on levels of haemoglobin and other biochemical markers would clarify the risks and benefits of different administration frequencies.

Two studies included in this review have made this comparison [16, 18]. In the first, the authors concluded that in children with moderate to severe anaemia, daily administration more effectively increases haemoglobin concentration and reduces anaemia [18]. The second study found that haemoglobin levels at the end of the intervention were higher in the non-daily regimes, with a greater reduction in the prevalence of anaemia and a higher percentage of children who remained non-anaemic [16].

Other studies in the literature have also made this comparison [24, 25] and concluded that providing MNP weekly with 30 mg of elemental iron produced haematological effects similar to daily administration with 12.5 mg of elemental iron in anaemic children from 6 to 23 months of age [24] as well as in non-anaemic school-age children [25].

More studies are necessary for comparing risks and benefits of different administration frequencies of MNP sachets. However evidences in this review lead us to suggest that public policies and programs with no anaemic children adopt flexible or non-daily administration model because it has greater adherence by children and caregivers and thus it is more effective and produce haematological effects similar to daily model.

Only one study, conducted by Geltmann et al. concluded that MNP had poor adherence, ranging from 30 % to 45 %. It is necessary to remember that this conclusion was based on a definition of adequate adherence as the consumption of five to seven sachets per week, a level that was considered a satisfactory range in all of the other studies [11]. However, satisfactory acceptability was hindered by several factors including inappropriate use of the sachets. In some studies, mothers reported refusal of MNP by children because of changes in the taste of food. MNP is formulated not to alter the organoleptic characteristics of food. It is possible that lack of knowledge led some mothers to use the MNP improperly, for example by mixing it with a liquid food, which is not recommended.

It is known that effectiveness of MNP depends of caregivers to be motivated to offer sachets MNP for children properly and without interruption. In this regard, additional efforts could be made to increase adherence and acceptability of the intervention. For example, high mineral concentrations in MNP sachets can be perceived by the taste; thus, careful attention must be given to the supplements’ sensory characteristics during the development process to minimize cases of rejection and to increase adherence to intervention. In addition, additional efforts should be planned in providing information and training to caregivers on appropriate use of MNP sachets since type of food used to mix them can affect its taste and consequently its acceptability.

One study cited the need for MNP to be mixed with food as a barrier to its use given the food situation in some of the countries in which these strategies have been implemented [23]. This study demanded public policies for food availability and distribution. As an alternative approach to address these issues, the Ministry of Health in Brazil developed the “NutriSUS” program, using fortification with MNP in young children attending day-care centres, in a sustainable and multisector strategy integrated with the cash transfer program “Bolsa Família.” [26].

Some studies reported that a lack of knowledge and experience with MNP generated concerns among caregivers about its use and safety [11, 22]. It is possible that these problems related to the product distribution model and the monitoring of children who participated in intervention, problems that could be resolved through training directed at health professionals and a greater dialogue between professionals and caregivers to address possible doubts and encourage caregivers about the importance of MNP in promoting health of their children. Furthermore, the reported concern and uncertainty of participants about the use of MNP is normal considering it is a new product. In the study conducted by Geltman et al. the mothers who completed the intervention had the greatest understanding about the importance of vitamins and minerals for their children [11].

The main side effects cited in these studies were diarrhoea, vomiting, and constipation, ranging 3-32 % of participants. These problems were more prevalent in the early days of MNP use. However, it must be cautiously interpreted when considering the possible side effects of MNP such as diarrhoea or other gastrointestinal diseases common in young children.

In a recent study by Soofi et al. in a malaria-endemic region of Pakistan with a stunting prevalence of 42 % among children under 5 years of age, the authors found strong evidence of an increase in the proportion of days with diarrhoea and an increase in bloody diarrhoea in the groups receiving MNP [20, 27]. That study found that MNP had a modest effect on micronutrient deficiencies, and very little effect on growth (*p* = 0 · 0017) [20].

 Another concern discussed in one study [23] is the need to mix MNP in semi-solid food because such food is not always available in low-income countries. The lack of knowledge and experience of professionals and caregivers in the use of MNP, the lack of availability of MNP, and its cost were also considered limitations in another study [22].

Despite of the described limitations, factors such as practicality of use increase in children’s appetite, weight gain, and increased activity level, among others, contributed to the high acceptability of MNP. In most studies, participants expressed an interest in continuing MNP use and recommended it to other mothers, which leads us to believe in the potential success of future interventions of home fortification with MNP.

Given these findings, the authors recommend that despite the demonstrated effect of MNP in preventing iron deficiency anaemia, future studies on home fortification with MNP should pay special attention to the definitions and strategies for monitoring adherence and acceptability . This evaluation should account for issues affecting adherence and acceptability of MNPs, highlighting once again the need to direct strategies to the particular characteristics of the target population. Acceptability rates for MNPs have generally been above 83 % in studies in developing countries, although rates are somewhat lower in studies performed in developed countries such as Canada (60 %) [ 1]. Because the use of the MNP requires their addition to foods suitable for young children, the home fortification strategy offers an opportunity to promote healthy complementary feeding, further improving the benefits of fortification [6]. However, it is likely that increased adherence to the use of MNP could result in greater impact on child health outcomes in future studies. Both adherence and acceptability can be increased when providing better guidance to caregivers and integration with the actions already performed by the healthcare team [6].

## Conclusions

This systematic review suggests that home fortification of complementary feeding with MNP has good adherence and acceptability. However, both adherence and acceptability could be increased with adjustments in the distribution model, such as better guidance to caregivers, and in MNP administration frequency. Special attention is necessary when monitoring MNP use in malnourished populations with a high incidence of diarrhoea. Additionally, more studies are needed examining the efficacy of MNP related to adherence and acceptability to compare the positive and negative aspects of various intervention models, for the success of future programs and public policies.

### Ethics approval and consent to participate

Not applicable. This review was reported in accordance with the PRISMA (Preferred Reporting Items for Systematic Reviews and Meta-Analyses) statement.

## Abbreviations

MNP: multiple micronutrients in powder
